# Stereotactic Laser Ablation for Medically Intractable Epilepsy: The Next Generation of Minimally Invasive Epilepsy Surgery

**DOI:** 10.3389/fsurg.2016.00064

**Published:** 2016-12-05

**Authors:** Michael J. LaRiviere, Robert E. Gross

**Affiliations:** ^1^Department of Radiation Oncology, University of Pennsylvania, Philadelphia, PA, USA; ^2^Departments of Neurosurgery and Neurology, Emory University School of Medicine, Atlanta, GA, USA

**Keywords:** epilepsy, stereotactic, minimally invasive, laser ablation, magnetic resonance thermal imaging

## Abstract

Epilepsy is a common, disabling illness that is refractory to medical treatment in approximately one-third of patients, particularly among those with mesial temporal lobe epilepsy. While standard open mesial temporal resection is effective, achieving seizure freedom in most patients, efforts to develop safer, minimally invasive techniques have been underway for over half a century. Stereotactic ablative techniques, in particular, radiofrequency (RF) ablation, were first developed in the 1960s, with refinements in the 1990s with the advent of modern computed tomography and magnetic resonance-based imaging. In the past 5 years, the most recent techniques have used MRI-guided laser interstitial thermotherapy (LITT), the development of which began in the 1980s, saw refinements in MRI thermal imaging through the 1990s, and was initially used primarily for the treatment of intracranial and extracranial tumors. The present review describes the original stereotactic ablation trials, followed by modern imaging-guided RF ablation series for mesial temporal lobe epilepsy. The developments of LITT and MRI thermometry are then discussed. Finally, the two currently available MRI-guided LITT systems are reviewed for their role in the treatment of mesial temporal lobe and other medically refractory epilepsies.

## Introduction

Epilepsy is a common neurological illness characterized by recurrent seizures. With an estimated lifetime prevalence of 3% and an often debilitating natural history (1), it is imperative that effective treatments are provided to minimize seizure burden and their medical, psychological, social, and economic implications. While first-line treatment of epilepsy draws from a broad pharmacopeia of anticonvulsant drugs (2), complete seizure control evades as many as one-third of medically treated patients (3). The International League Against Epilepsy task force defines drug-resistant epilepsy as lack of seizure freedom despite two separate antiepileptic drug regimens (4). Mesial temporal sclerosis (MTS), in particular, has been observed to be associated with drug resistance (5).

For these patients, surgery is highly effective in controlling and often abolishing seizures (5). The mainstay of surgical treatment has been open brain resection including anterior temporal lobectomy (ATL) and selective amygdalohippocampectomy (SAH), both of which include several variant approaches (6–11). However, to further improve the safety profile of surgery (8), over a half century of effort has been directed at developing minimally invasive surgical techniques for treating medically refractory epilepsy with defined epileptogenic foci. The present review discusses the development of minimally invasive approaches, beginning with the original stereotactically guided ablation techniques, modern imaging-guided stereotactic radiofrequency (RF) ablation, and the emerging development of imaging-guided stereotactic laser ablation.

## Open Temporal Lobe Resection

Open ATL has been a well-established treatment for over half a century (6, 7). Wiebe et al. demonstrated 64% seizure freedom at 1 year in operated patients (58% in the intent-to-treat surgery group, but which included several non-operated patients) in a randomized controlled trial of ATL vs. best medical therapy (9). A recent meta-analysis of 11 trials reported 1-year seizure freedom rates of 75% following ATL (*n* = 620) (note this and the numbers below from this study are a weighted average and cannot be calculated simply from the numbers presented) (12). The subset with hippocampal sclerosis (AKA MTS) yielded higher rates (78%, *n* = 535). Similarly, although low accrual halted it prematurely, the recent early surgical therapy for drug-resistant temporal lobe epilepsy (ERSET) randomized controlled trial of ATL vs. continued medical therapy within 2 years of intractability found that surgery yielded freedom from disabling seizures in 11 of 15 (73%) patients, as compared to 0 of 23 medically treated patients (10). While ATL does carry a risk of decline in verbal function such as naming, particularly with more extensive lateral resections (11), on the whole it is relatively safe. The 2001 randomized controlled trial underscores this point: with 40 patients assigned to medical treatment and 40 assigned to surgery, the surgery group saw one postoperative stroke causing a neurologic deficit that fully resolved, one ventriculoperitoneal shunt placement for bleeding-induced communicating hydrocephalus, and no deaths (9). By comparison, the medical arm saw three episodes of status epilepticus in two patients, and one death due to presumed sudden unexplained death due to epilepsy (SUDEP) (9).

The advent of more selective approaches sparing resection of the anterior temporal lobe dates back to the mid-20th century (13–15), the goal of which is in part to minimize potential neuropsychological impact from the temporal resection. Although seizure-free rates were long thought to be similar to ATL, the recent meta-analysis, which included 583 patients having undergone SAH, reveals a slightly lower effectiveness of 67% (*n* = 557), and 71% of those patients with MTS (*n* = 557) becoming seizure free (12). Statistically, for every 13 patients treated with SAH, one who would not become seizure free otherwise would with an ATL.

However, the question of whether selective AH spares neuropsychological functions is not straightforward. On the basis of a large number of comparative (albeit non-randomized) studies, using various different measures over many years, Helmstaedter recently concluded that “selective surgery is clearly preferred (16).” As one example, patients with MTS undergoing left-sided ATL experienced decreased verbal learning as compared to SAH patients (while both experienced losses in long-term memory) (17, 18). However, selective approaches – while preserving tissue, such as the temporal pole, from resection – subject fibers of passage to risk from dissection. In this regard, Helmstaedter, in a group of 97 seizure-free patients following either a temporal pole resection and AH vs. trans-Sylvian selective AH, found that verbal memory was better after left-sided temporal pole resection compared to trans-Sylvian selective AH, whereas on the right side, figural memory was better after the selective approach as compared to the temporal polar resection (19). While suggesting that the temporal pole and stem have distinctive roles in figural and verbal memory, respectively, the implication for open surgery is that compromise of neuropsychological functions can occur following both resective and dissective approaches through the temporal lobe during amygdalohippocampectomy. Helmstaedter (2013) concluded “no single surgical approach may be discerned to be the safest with regards to cognition, irrespective of seizure control. According to the surgical approach, different tissues and fiber tracts that hinder the surgical procedure or locate to adjacent areas may be affected and, either way, this has consequences for cognition (16).”

These effects on cognitive functions have, among other reasons, and despite the satisfactory rate of seizure freedom, driven interest in minimally invasive techniques to further improve safety and tolerability of the surgery.

## Stereotactic Ablation

### Original Trials

Initially mainly developed as a treatment for movement disorders involving the basal ganglia (20), ablative techniques rely on stereotactic guidance to create a permanently destructive lesion. Whereas deep brain stimulation requires chronic implantation of electrodes (21–23), stereotactic ablation is an acute procedure that carries a lower risk of chronic complications, such as infection, than chronic instrument implantation. However, an advantage of deep brain stimulation is that stimulation can be adjusted or turned on and off.

Stereotactic ablation of epileptogenic foci was first described over 50 years ago by Narabayashi and colleagues in Tokyo (24). In a series of 60 patients, 46 of whom were diagnosed with epilepsy and 14 of whom had EEG disturbances or behavioral symptoms, the amygdala was stereotactically targeted using pneumoencephalographic X-ray imaging and electrophysiologic recording for guidance. To confirm placement in the amygdala, the authors recorded the neuronal spike pattern upon giving the patient ether for olfactory stimulation. The target was ablated with an oil-wax-lipiodol contrast dye mixture. Ultimately, they saw improvement in 85% (51/60) of patients, substantial improvement in 48% (29/60), and no change in only 3% (2/60). Morbidity was very low: one patient experienced a temporary palsy for 3 weeks and one patient developed several days of hypersexual behavior.

Despite this promising report, subsequent small trials in the 1960s yielded seizure freedom in just 19–30% of patients (Table 1) (25, 26). Notably, in this decade a −120°C cryoprobe was developed for stereotactic cryoablation of the amygdala, as reported by Heimburger et al. (26). Stereotactic ablation in the 1970s produced seizure freedom rates as high as 63% in small series (27, 28), and rates of seizure improvement (including seizure freedom) ranged from 50 to 100% (27, 29–40). Although there was a paucity of published reports in the 1980s, one series of 70 patients undergoing medial amygdalotomy and anterior hippocampotomy^1^ found seizure improvement in 86%, including seizure freedom in 11% (41). Taken together, the early development of stereotactic ablation saw a number of centers try several variations on the technique, with variable results. Later, developments in CT and MR imaging would allow for major improvements in such minimally invasive treatments for epilepsy.

**Table 1:** **Summary of stereotactic ablation trials**.

Study	Seizure free			Improved only			Improved + seizure free			Target
	#	Total	%	#	Total	%	#	Total	%	
**Tokyo**										
Narabayashi et al. (24)	–	60	–	51	60	85	51	60	85	A
Narabayashi and Mizutani (33)	9	25	36	8	25	32	17	25	68	A
**Boston**										
Schwab et al. (25)	3	10	30	4	10	40	7	10	70	A
**Paris**										
Talairach and Szikla (37)	9	14	64	2	14	14	11	14	79	AH
**Freiburg, Germany**										
Umbach (38, 39)	5	18	28	11	18	61	16	18	89	AH
Bouchard and Umbach (29)	–	50	–	33	50	65	33	50	65	
**Wurzburg, Germany**										
Schaltenbrand et al. (36)	–	5	–	3	5	60	3	5	60	
**Indianapolis**										
Heimburger et al. (26)	4	21	19	12	21	57	16	21	76	A
Heimburger et al. (31)	–	58	–	29	58	50	29	58	50	
**Copenhagen**										
Vaernet (40)	5	27	19	10	27	37	15	27	56	A
**Bratislava, Slovakia**										
Nadvornik and Sramka (27)	5	8	63	3	8	38	8	8	100	H
**Durham, NC, USA**										
Flanigin and Nashold (30)	4	16	25	6	16	38	10	16	63	AH
**Warsaw**										
Mempel et al. (41)	8	70	11	52	70	74	60	70	86	AH
**Omaha, NE, USA**										
Patil et al. (42, 43)	–	3	–	3	3	100	3	3	100	AH
**London, ON, Canada**										
Blume et al. (44)	6	14	43	4	14	29	10	14	71	AH
Parrent and Blume (34)	2	19	11	8	19	42	10	19	53	
**Birmingham, AL, USA**										
Kuzniecky and Guthrie (58)	3	8	38	4	8	50	7	8	88	HH
**Tianjin, China**										
Yang et al. (45)	10	23	43	11	23	48	21	23	91	AH
**Prague**										
Kalina et al. (46)	12	16	75	3	16	19	15	16	94	AH
Malikova et al. (48)	14	17	82	3	17	18	17	17	100	AH
Liscak et al. (49)	25	32	78	5	32	16	30	32	94	AH
Malikova et al. (50)	19	26	73	5	26	19	24	26	92	AH
Malikova et al. (52)	27	35	77	5	35	14	32	35	91	AH
Malikova et al. (53)	28	37	76	7	37	19	35	37	95	AH
Vojtech et al. (54)	43	61	70	6	61	10	49	61	80	AH
**Niigata, Japan**										
Homma et al. (59)	3	5	60	2	5	40	5	5	100	HH
Kameyama et al. (60)	19	25	76	6	25	24	25	25	100	HH
**Lyon, France**										
Catenoix et al. (47)	1	41	2	20	41	49	21	41	51	TL, PL, insula, SMA, OL, FL, C, PSC
**Visualase: Houston, TX, USA**										
Curry et al. (98)	5	5	100	–	5	–	5	5	100	MTL, FL, C, HH
Wilfong and Curry (117)	12	14	86	2	14	14	14	14	100	HH
Esquenazi et al. (125)	2	2	100	–	2	–	2	2	100	PVNH
**NeuroBlate: St. Louis, MO, USA**										
Hawasli et al. (113)	1	1	100	–	–	–	1	1	100	insula
**Beijing**										
Wu et al. (56)	4	7	57	0	7	0	4	7	57	MTL
**Parma, Italy**										
Cossu et al. (57)	16	89	18	9	89	10	27	89	28	TL, FCD, nodular heterotopy
**Visualase: Cleveland, OH, USA**										
Gonzalez-Martinez et al. (124)	–	1	–	–	1	–	–	1	–	PVNH
**Visualase: Atlanta, GA, USA**										
Willie et al. (99)	7	13	54	3	13	23	10	13	77	MTL
Willie and Gross (101)	5	5	100	–	–	–	5	5	100	MTL
McCracken et al. (132)	4	5	80	–	–	–	4	5	80	TL, OL, FL
**Visualase: Miami, FL, USA**										
Lewis et al. (129)	7	17	41	1	17	6	8	17	47	FCD, TS, HH, MTL, other
**Visualase: Philadelphia, PA, USA**										
Kang et al. (122)	14	20	70	4	20	20	18	20	90	MTL

*A, amygdala; AH, amygdala-hippocampus; H, hypothalamus; HH, hypothalamic hamartoma; TL, temporal lobe; MTL, mesial temporal lobe; PL, parietal lobe; OL, occipital lobe; FL, frontal lobe; SMA, supplemental motor area; PSC, primary sensory cortex; C, cingulate; PVNH, periventricular nodular heterotopia; FCD, focal cortical dysplasia; TS, tuberous sclerosis*.

### Imaging Guidance for Radiofrequency Ablation

In 1995, image-guided targeting for epilepsy ablation was revolutionized with the report of the first CT-guided stereotactic amygdalohippocampotomy (42, 43). Two years later, Blume et al. reported a series of 14 patients with MRI targeting for RF ablation of the amygdala and hippocampus *via* a lateral (or orthogonal) approach. At 9–31 months follow-up, they reported that six (43%) were seizure free (after initial seizures in three), but they did not specify the length of time of seizure freedom. The results seemed to show a relationship to whether they performed confluent vs. discrete lesions (44). However, the subsequent report on the larger series of 19 patients in 1999 showed seizure freedom in just two patients (11%) (34). Whereas only one of five patients receiving discrete lesions (involving 13–21 mm, mean 16.8 mm, of hippocampus) experienced favorable seizure improvement (but not seizure freedom), 9 of 15 patients receiving confluent lesions (15–34 mm, mean 21.5 mm) experienced favorable outcomes; only two, however, became seizure free. Morbidity was very low, with just one patient developing an asymptomatic hematoma along the electrode track. These reports suggested that RF ablation could be effective, but that greater extent of ablation was likely a critical factor.

In the next several years, different groups achieved highly variable results, ranging from 2 to 75% seizure freedom (45–47). Whereas Yang and colleagues reported 43% (10/23) seizure freedom (45), and Kalina et al. reported 75% (12/16) (46), Catenoix et al. felt that their 2% (1/41) seizure freedom was so disappointing that they recommended stereotactic ablation be used only when resection could not be performed (47). Importantly, unlike prior trials, Catenoix’s series showed that RF ablation could also be used to target structures in the neocortex.

Although some of these results were disappointing, recent work has rekindled interest in MRI-guided stereotactic RF ablation. In a series of 17 patients, Malikova et al. achieved seizure freedom in 82% and improvement in the remaining 18% at 2-year follow-up (48). This group took an occipital approach to the long axis of the hippocampus and amygdala, and used a “string electrode” to perform radially distributed lesions. This allowed greater thermal coverage in the coronal plane as well as along the long axis: 16–38 lesions (median 25) produced ablations spanning 30–45 mm (median 35 mm) (49). Indeed, by using MRI to analyze the target structure’s size decrease following the procedure, they were able to correlate their imaging analysis with clinical outcome (48). Not surprisingly, they found that decreased amygdala–hippocampal volumes were associated with better seizure control. Subsequently, Liscak et al. reported that among 51 patients, 78% achieved seizure freedom and 16% had seizure improvement over at least 2-year follow-up (49). Aside from such transient side effects as headache, meningitis, and upper meningeal syndrome [also seen in the authors’ prior trials (46, 48)], four patients developed hematomas, and one of these patients developed obstructive hydrocephalus that resolved with drainage. In 2011, Malikova et al. reported seizure freedom in 73% and seizure improvement in another 19% of 26 patients (50). Whereas Vojtech et al. in 2012 concluded that, among 31 patients, RF amygdalohippocampectomy caused no significant deterioration in memory and cognitive parameters (51), Malikova et al., in that year, reported significant verbal and long-term memory improvements following ablation of right-sided lesions, with greater cognitive improvements seen with greater hippocampal volume reduction (52). In the latter study, they reported 77% seizure freedom among 35 patients at 1 year. In 2013, Malikova et al. reported 2-year seizure outcomes among 37 patients, reporting 76% seizure freedom (53). In contrast to their 2012 short-term data, in their 2013 work, they found significant improvements in global, verbal, semantic long term, and working memory, as well as delayed recall and attention in both right-sided and left-sided lesions. The following year, Vojtech and colleagues reported long-term data for 61 patients with an average follow-up of over 5 years, with 70% Engel class 1 (54). Reported adverse events included four hematomas, one case of hydrocephalus requiring shunt placement, three cases requiring open surgery, two patients who underwent repeat RF ablation, two patients with meningitis, and six new psychiatric diagnoses including one suicide. At the time of this publication, Malikova and colleagues’ most recent report in 2015 looked at cognitive outcomes in 37 patients treated with RF ablation (55). Whereas Malikova et al. in 2012 found that better cognitive outcome correlated with increased *right* hippocampus volume reduction (52), their 2015 paper showed *worse* verbal memory performance with increased *left* hippocampal reduction (the majority of these patients had Wada testing-confirmed left-side speech and memory dominance). Taken together, these results suggest that the Prague center has been able to achieve seizure freedom in 70–82% of patients, and combined seizure improvement or freedom in 80–100% of patients (48–50). Clearly, these results were highly encouraging and have ignited renewed interest in stereotactic ablation (56, 57).

The past five decades of pioneering trials demonstrate the versatility of stereotactic ablation. This technique is able to target not only the mesial temporal lobe but also other neocortical and deep brain structures (58–60), and it produces an immediate effect on seizure control. The variety of modalities tested to deliver the ablative lesion – oil-wax, cryoablation, and RF – suggests that future research with novel ablation techniques, coupled with advancements in MRI imaging, are likely to further refine targeting precision and protection of critical structures.

## Development of MRI-Guided Stereotactic Laser Interstitial Thermotherapy

### Laser Interstitial Thermotherapy

In 1983, S. G. Bown published a seminal review surveying lasers used for tumor phototherapy (61). While most of the research until then had used CO_2_, argon, and neodymium:yttrium-aluminum-garnet (Nd:YAG) *external* lasers, in his review, he also reported his original results from Nd:YAG *interstitial* laser ablation in an excised malignant melanoma model (61). By extrapolating the results of his prior external beam work, Bown hypothesized that interstitial laser ablation would fibrose, rather than perforate tissue (61, 62). Four years later, Matthewson et al. reported that interstitial Nd:YAG thermocoagulation in the rodent liver indeed yielded reproducible volumes of coagulative necrosis and devascularization that were ultimately replaced by granulation tissue and fibrosis (63). With the goal of real-time imaging to further refine targeting, in 1989, Steger et al. used ultrasound in a series of five human patients with various extracranial tumors (64). This allowed them to visualize, in real-time, tumor necrosis resulting from their Nd:YAG laser ablation. Nd:YAG laser ablation was further tested in such sites as the canine prostate (65), rodent lung parenchyma (66), and human liver metastases (67).

In 1990, laser interstitial thermotherapy (LITT) was first reported for intracranial tumors, yielding 100% regression in five human patients with gliomas or brain metastases (68). Several other groups soon reported similar results in patients with grades II–III gliomas (69–71); postoperative MRI and histologic analysis revealed well-demarcated volumes of coagulative necrosis (70, 71). As Roux, Bown, and colleagues reviewed in 1996, these histological findings are consistent with a pattern of tissue damage unique to laser thermocoagulation: immediately adjacent to the Nd:YAG laser fiber, a small fibrin-filled cavity is surrounded by a larger volume of dense coagulation, itself encased in a shell of loose coagulation, ultimately transitioning to edema and then normal brain tissue (72). In a similar vein, Schwabe and colleagues defined five discrete regions by comparing pre-contrast T1-, post-contrast T1-, and T2-weighted MRI: a T2-intense catheter track, a T1 pre- and post-contrast-intense central zone, a T2-intense peripheral zone, a T1 post-contrast-enhancing peripheral zone border, and T2-intense surrounding edema (73). After nearly 4 years follow-up of 18 patients, they found that while laser ablation caused lesions initially to *increase* in size, they eventually lost half their volume in approximately 3 months and then exponentially decreased in size.

Ultimately, the first two decades of LITT saw the development of a potentially promising technique for both extracranial and intracranial tumors. But, its use in functional neurosurgery, for smaller targets near eloquent structures, would demand advances in real-time monitoring of tissue damage.

### MRI Thermometry

The development of MRI thermal imaging (MRTI) began in 1988 when Jolesz et al. used MRI to detect reversible tissue changes below 60°C, and coagulation from 60 to 100°C following laser ablation in the rabbit brain (74). It was later determined that temperatures between 45 and 90°C cause irreversible coagulation, with cavity formation above 100°C (72, 75). On the other hand, Hayat and Friedburg found that tumor cells may be more thermosensitive, susceptible to damage at temperatures as low as 42.5–45.5°C (72, 76). Thus, the development of MRI thermal monitoring has sought to optimize measurement up through 100°C, as well as in the sub-45°C range that may affect tumor cells but which critical structures must not exceed (77).

In 1991, Ascher and colleagues reported that real-time MRI could detect changes in tissue characteristics 3 min after initiating laser ablation in brain tumors (78). The following year, Fan et al. undertook a phase I clinical trial using real-time MRI thermometry of Nd:YAG ablation in three patients with brain tumors (79). By first calibrating T1 signal change with probe-measured temperature change in laser-treated cadaver brains, MRI thermometry could then be used with the three patients. However, this T1-based method was limited by a non-uniform RF field susceptible to external interference, potentially interfering with the accuracy of measured temperature change. A similar trial was later performed in eight patients, achieving reproducible changes in T1-weighted signal intensity during laser therapy (80). Here a T1-weighted 2D-fast low angle shot (2D-FLASH) sequence, with 15 s acquisition time, delivered viewable images with a temporal resolution of 30 s. Signal changes were seen as early as 1 min, but again at an average of 3.1 min after the start of laser therapy. As one might expect, signal change evolution was different immediately adjacent to versus more peripherally around the laser fiber tip, reflecting differential tissue changes in these two zones (80), consistent with prior studies (72).

While other groups continued to use modified FLASH sequences for MRTI in LITT (81, 82), Fried et al. (83) and Matsumoto et al. (84, 85) used a different sequence to better monitor temperature in both phantoms and various animal tissues (83–85). In 1992, a T1-weighted rapid acquisition with relaxation enhancement (RARE) sequence was developed to achieve an improved temporal resolution of 16 s, temperature resolution of less than 6°C between pixels, and linear signal change from 10 to 50°C (85). Subsequently it was shown that, among T1-weighted sequences, fast spin-echo with a relaxation time (TR) of 100 ms delivered both improved temperature change sensitivity of less than 1.5% signal change per degree celsius, and acquisition time as fast as 5 s (84). Despite these advances in MRTI, thermotherapy-induced tissue change could still cause tissue-dependent signal change that would interfere with accurate temperature monitoring (83).

To address these limitations, water proton resonance frequency shift MRTI was developed (86), and this is the technique used with modern laser ablation systems (87–92). After demonstrating its feasibility in agarose gel phantoms with 0.2°C accuracy (86, 93), De Poorter et al. applied the technique to human muscle *in vivo* (93). In 1998, Kettenbach and colleagues built on this work by treating seven liver and three brain neoplasms using an MRI software interface to estimate temperature change in real time (77). They compared three techniques for estimating temperature and found that the above-described water proton chemical shift phase mapping (86, 93) proved to be the most sensitive technique for mapping temperature change (77). Eventually, various groups adopted the water proton chemical shift phase mapping technique (94–96), and this sequence would ultimately enable the development of such modern MRI-guided laser ablation systems as Visualase (87, 91, 92) and NeuroBlate (88–90).

## Modern MRI-Guided Stereotactic Laser Interstitial Thermotherapy Systems

Like RF ablation, MRI-guided LITT (MRIgLITT) is able to reach deep structures that would be inaccessible by open craniotomy and resection. Yet, compared with RF ablation, MRIgLITT has the advantages of real-time imaging and monitoring of thermal dose delivery using an Arrhenius equation-based model (92, 97). Moreover, MRIgLITT can achieve a larger ablation volume that may require fewer probe targets and placements (Table 2). Disadvantages of this system are the potential for cerebrospinal fluid and blood flow to divert heat away from the target zone, although this is true for RF as well. However, users have felt that the ablation volume does adequately conform to the target lesion, and that encapsulated lesions seem to “contain” the heat (98, 99). MRIgLITT provides an unparalleled level of targeting precision: for instance, compared with open resection, MRIgLITT causes significantly less naming and object recognition decline (100), and repeat ablation in select patients with seizures refractory to initial laser ablation has been effective and well-tolerated (101).

**Table 2:** **Comparison of radiofrequency ablation and MRI-guided laser interstitial thermotherapy**.

	RF ablation	MRIgLITT
Target structures	Can target deep structures	Can target deep structures
Temperature control	Only at tip	At tip and beyond
Visualization of lesion formation	No	Yes
Lesion size	Empirically determined	Preplanned and adapted intraoperatively
Thermal spread	Difficult to control	Software controlled
Transition zone	Large	Small

### Commercial LITT Systems

#### Visualase

**Figure 1:**
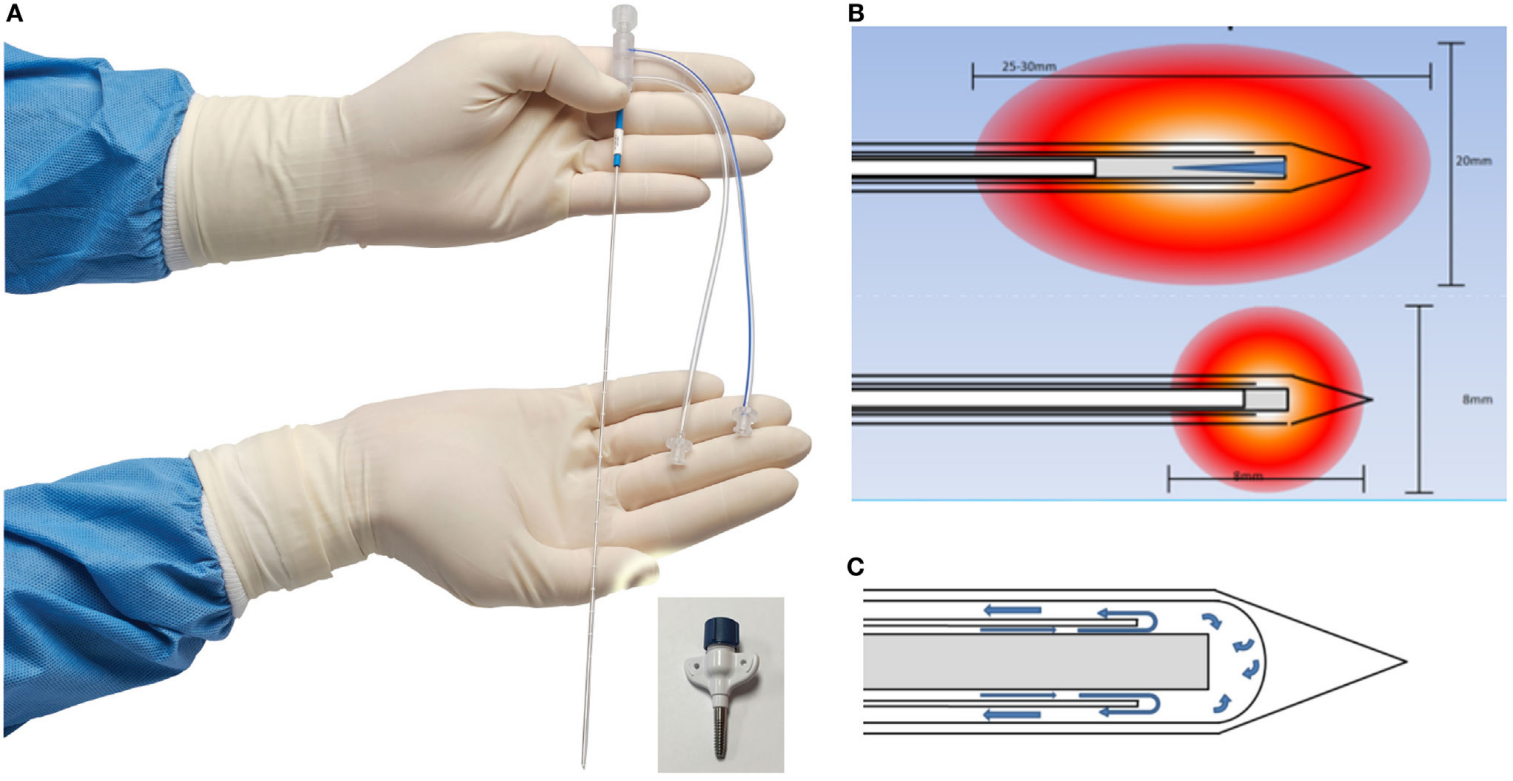
**Visualase Cooled Laser Applicator System (VCLAS)**. **(A)** The cooled laser applicator. The ports are for inflow and outflow of saline coolant. Inset: titanium anchor for insertion. **(B)** VCLAS with 10 mm (top) and 3 mm (bottom) diffuser tips and simulated ablation zones. The VCLAS is a two-channel cannula: the optical fiber with diffuser tip is passed through the central channel, whereas the coolant circulates through the outer channel, as depicted in **(C)**. Images provided by Medtronic, Louisville, CO, USA.

**Figure 2:**
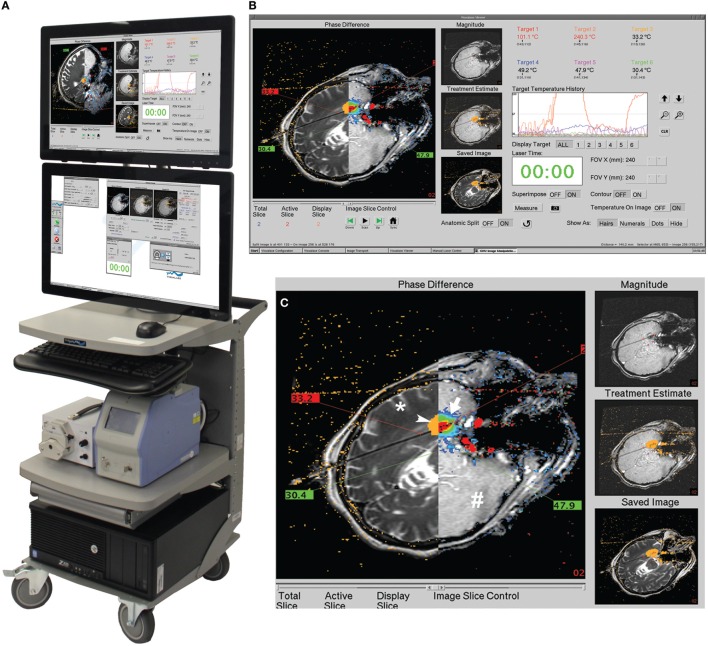
**Visualase workstation**. **(A)** Entire workstation is shown, consisting of the computer and two monitors (at top), the 15 W, 980 nm diode laser (bottom right) and the peristaltic pump for the saline coolant (bottom left). **(B)** The top monitor screen is shown and magnified in **(C)**, with the axial image plane being visualized. The screen can be quickly toggled between two or three chosen image planes, as desired; moreover, one plane can be visualized on the top monitor and concurrently a different one on the bottom monitor. The left side of the top monitor is a sliding window that can depict the four types of information being acquired all at once: phase image (arrow), i.e., “thermal map”; magnitude image (^#^) – a rapidly acquired but low-resolution anatomical image from the same sequence (fast spoiled gradient echo, SPGRE) that produces the phase image; irreversible damage zone estimate (arrowhead), calculated from the Arrhenius equation; and high-resolution anatomical image (*) in same plane as the phase image, acquired prior to the phase mapping. Also shown in sliding window are temperatures of the user-defined safety points. Images provided by Medtronic, Louisville, CO, USA.

**Figure 3:**
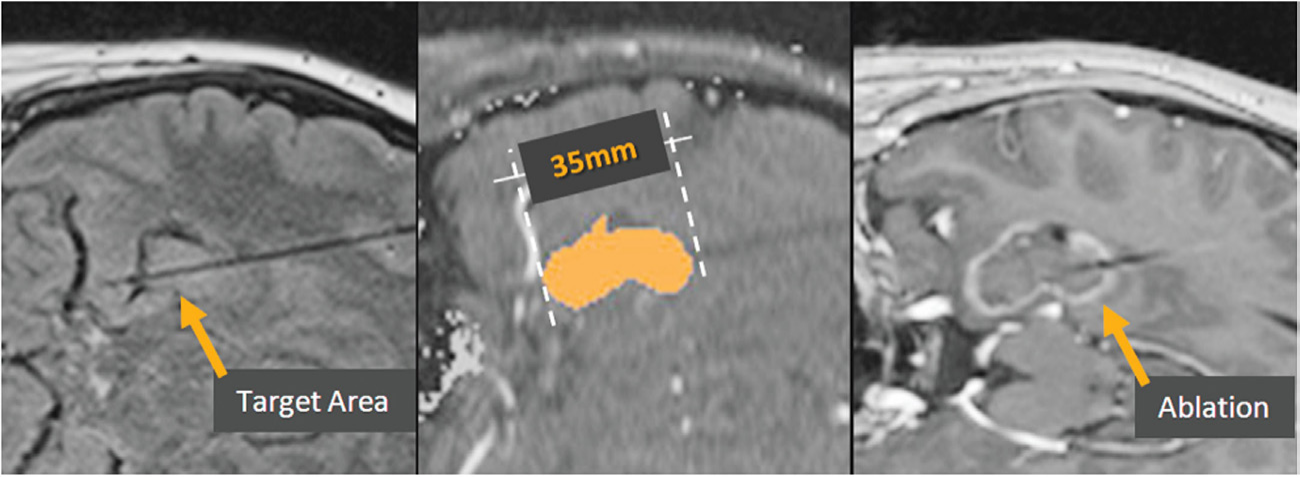
**Visualase ablation**. Left, target region in the hippocampus, with Visualase cooling catheter laser applicator in place. Center, irreversible damage zone estimate (red) during the laser ablation. Right, post-ablation contrast-enhanced T1 image depicting the actual damage zone surrounded by region of blood–brain barrier breakdown (contrast enhancement).

Visualase is an MRIgLITT system, FDA approved in 2007 “to necrotize or coagulate soft tissue through interstitial irradiation or thermal therapy under (MRI) guidance in… general surgery,… neurosurgery,… and urology….” The Visualase Cooled Laser Applicator System (VCLAS) uses a saline-cooled catheter to deliver a fiber optic with a 3 or 10 mm diffusing tip (Figure 1). Saline coolant is circulated through the VCLAS by a peristaltic pump (Figure 2). Laser energy is delivered through a 15 W, 980 nm diode laser, which is controlled *via* a user monitoring station that provides user-defined safety limits that trigger automatic laser shutoff when exceeded (Figure 2) (98). MRTI monitoring is provided in up to three planes, transferred to the monitoring station from the MRI by an FTP-like protocol (Figure 2) (92). The software uses an Arrhenius rate process model of thermal damage to depict the “irreversible damage zone” estimate, based on the time–temperature relationship (92, 102). The software further allows for the definition of up to six user-defined thermal safety limits that, when reached, trigger automatic shutdown of the laser. Typically, three markers are placed near the laser fiber to prevent the temperature from exceeding 90°C, which could damage the fiber and/or lead to a steam event, and three markers are placed at nearby tissue at risk with a limit of ~50°C to prevent off-target thermal effects. The laser fiber assembly is typically implanted through a plastic or titanium anchor bolt (Figure 1A, inset) that is stereotactically implanted in the bone using one of a variety of stereotactic techniques, either classic frame-based or more recent “frameless” techniques. After insertion of the cooling-cannula/laser fiber assembly, a planning MRI is used to select the target volume for ablation. The extent and temperature of the thermal delivery is assessed in near real time, after which contrast-enhanced T1 imaging, diffusion weighted imaging, and other protocols demonstrate the acute lesion volume (Figure 3) (98, 99).

McNichols and colleagues tested the Visualase system in porcine and canine brain models (92). By specifying temperature thresholds and their associated geometric volumes with real-time MRI imaging, they were able to define both a target for thermal ablation and critical structures that would be protected from high temperatures. The software feedback successfully achieved the desired temperatures in the ablation target, and the irreversible damage prediction models closely correlated with histologic findings.

#### NeuroBlate

**Figure 4:**
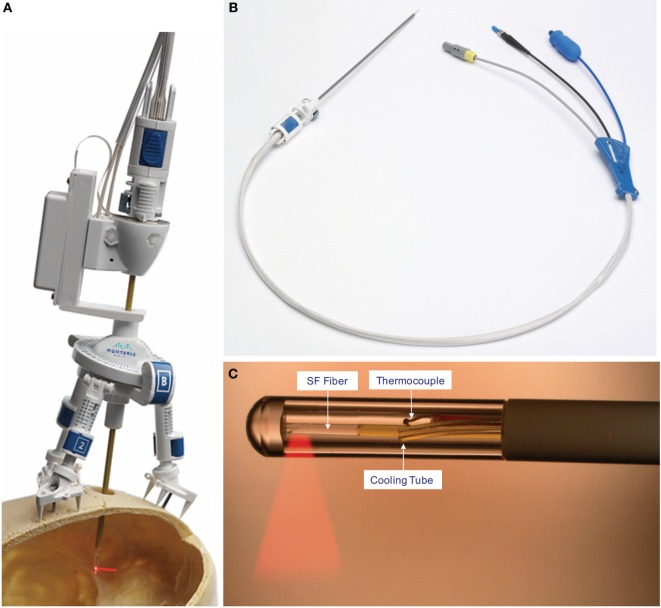
**NeuroBlate catheter**. **(A)** The AXiiiS stereotactic miniframe is attached to the skull with spikes and bone screws. **(B)** The NeuroBlate 12 W, 1064 nm Nd:YAG laser delivery probe has a depth stop adjustment with a locking interface. **(C)** The SideFire fiber emits laser energy through a clear sapphire lens at a 78° angle. CO_2_ gas cools the tip and the thermocouple transmits temperature data to the M*Vision software. FullFire diffusing, rather than directional, tip available (not shown). ©Monteris Medical. Used by permission. The use of any Monteris Medical photo or image does not imply Monteris’ review or endorsement of any article or publication.

**Figure 5:**
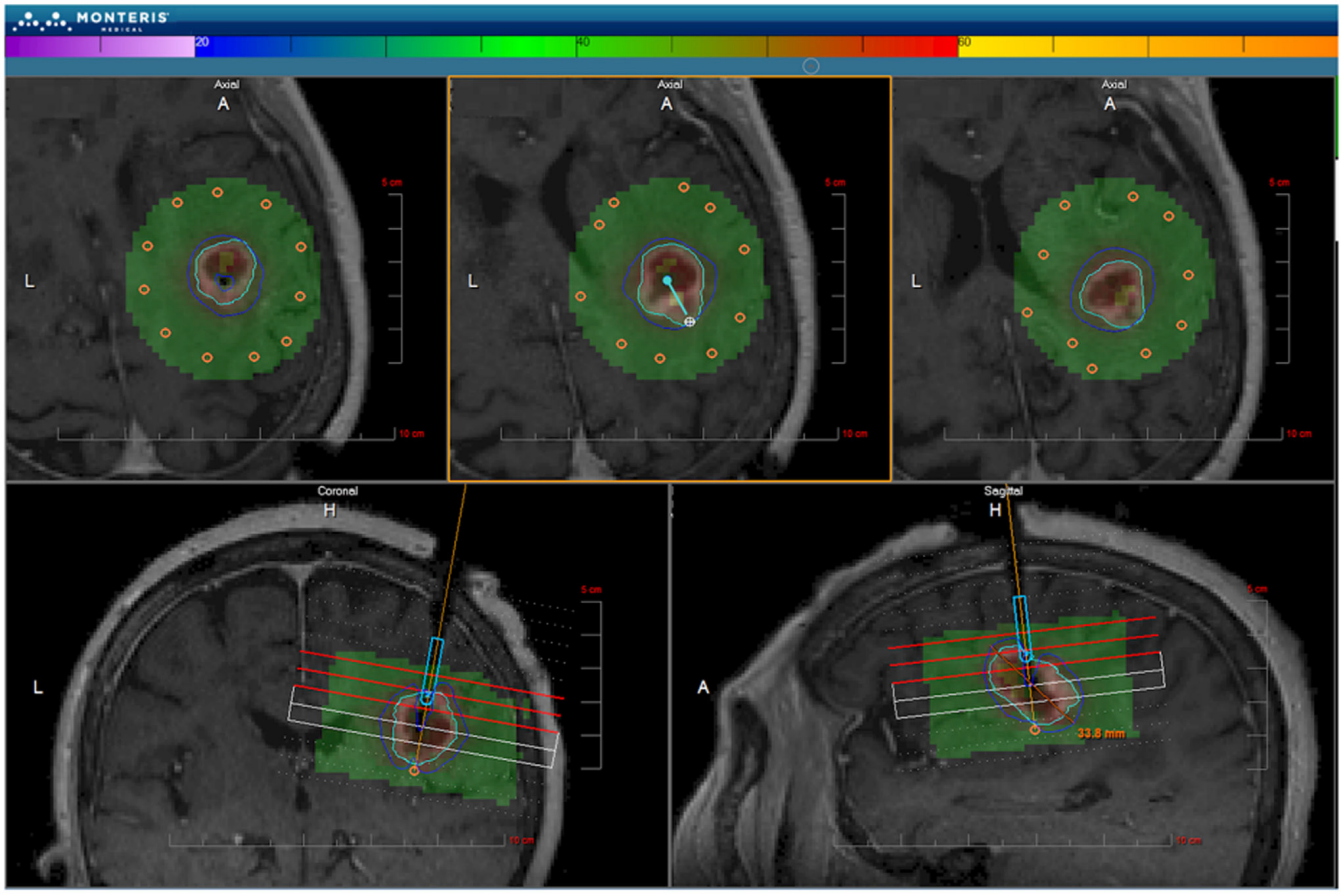
**NeuroBlate software**. The M*Vision software allows the target volume to be contoured slice-by-slice, thermal dose threshold lines are calculated, and the laser probe trajectory can be planned. After planning, the software executes the treatment allowing the user to monitor real-time thermal imaging. Laser adjustments can be made during treatment. Once the ablation is complete, MR images are obtained and compared with thermal dose thresholds. ©Monteris Medical. Used by permission.

NeuroBlate, originally called AutoLITT, is an MRIgLITT system that received FDA clearance in 2009 for intracranial and extracranial use (88). NeuroBlate uses a CO_2_-cooled catheter with a 12 W, 1064 nm Nd:YAG laser (Figure 4). Initially, the system was only offered with a “side-fire” tip that ostensibly distributes the laser energy asymmetrically; it is now offered with a 10 mm diffusing tip for more symmetric heating. The NeuroBlate software allows for preoperative designation of the target lesion with MR imaging and estimates thermal ablation volume intraoperatively. The system plans ablation on a slice-by-slice basis: once the laser is placed, intraoperative MRTI begins, the software appropriately orients the side-firing port, and thermal damage is monitored by the surgeon (Figure 5). Once ablation is complete on a given slice, the software robotically advances the laser probe to the next slice location, begins MRTI, plans ablation for that slice, and again orients and fires the laser. The system proceeds with this sequence until completion. Because this laser has a damage range of 1.5 cm, repeat placements are needed for targets larger than 3 cm (88).

## Clinical Outcomes Using Commercial MRIgLITT

### Clinical Outcomes Using Commercial MRIgLITT for Tumor Treatment

The first human application of the present systems was in six patients with brain metastases treated using the Visualase system (91). Perioperative morbidity was minimal, and no tumor recurrence was seen within the ablation zone at 3 months. Moreover, postoperative MRI showed that ablation lesions were consistent with preoperative planning to within 6 mm. Since then, several groups have used Visualase in the treatment of primary brain tumors (103–106), brain metastases, radiation necrosis (104, 107–109), spinal metastases (110), and other tumors outside the CNS, most notably prostate cancer (111, 112). NeuroBlate was first trialed in 10 patients with recurrent glioblastoma (90) and has since been used in patients with primary gliomas (89, 113), brain metastases (113, 114), and radiation necrosis (115, 116). Further discussion is beyond the scope of this review.

### Clinical Outcomes Using Commercial MRIgLITT for Epilepsy Treatment

Curry et al. published the first experience with MRIgLITT in epilepsy in five pediatric patients in 2012 (98). Epileptogenic foci included one tuberous sclerosis (TS) lesion in the cingulate gyrus, one cortical dysplasia in the frontal lobe, two hypothalamic hamartomas, and one patient with both a sclerotic mesial temporal focus and a superior temporal neurocysticercosis calcification. In three of the five patients, the target lesion was completely ablated. All were seizure free at the last follow-up (2–13 months) but only two had reached 12 months – the “gold standard” (cingulate TS, MTLE). The published experience, which now covers a fair number of cases of various etiologies, will be discussed.

#### Hypothalamic Hamartomas

Following their initial report (98), Wilfong and Curry (117) published a series of 14 pediatric patients with hypothalamic hamartomas (HH) treated with MRIgLITT, achieving seizure freedom in 86% of patients at a mean of 9 months follow-up (range 1–24). Notably, safety was excellent with one patient experiencing an asymptomatic subarachnoid hemorrhage, and with a median length of stay of 1 day. Zubkov et al. reported a single case of a disabling amnestic syndrome following laser ablation of a HH in a 19-year old with a prior right temporal lobectomy (118). Post-ablation imaging showed edema in the bilateral mammillary bodies, which likely contributed to the deficit, although the role of the prior lobectomy is uncertain. Given the invasiveness of alternative therapies such as open resection (119) and delay to effectiveness and possible radiation-induced adverse effects from stereotactic radiosurgery (120), this is an important new treatment modality for this indication, but should be judiciously applied and studied.

#### Mesial Temporal Lobe Epilepsy

##### Outcomes

Willie et al. reported the use of MRIgLITT to perform stereotactic laser amygdalohippocampotomy (SLAH) in 13 adult patients with mesial temporal lobe epilepsy (99). Seven patients (54%) were seizure free at 5–26 months follow-up, and an additional three (23%) had seizure reduction. With the exception of one patient with an acute subdural hematoma that was evacuated without sequelae, given a lack of postoperative symptoms in the first three patients, the remaining patients did not require postoperative ICU care, and overall median hospital length of stay was just 1 day. Subsequently, Waseem et al. compared SLAH in seven patients >50 years of age to outcomes after SAH in seven patients (121). Four of the five SLAH patients with outcomes reported at 1 year or more were seizure free (they did not report the outcome on the other two patients), and all of the seven SAH patients were seizure free. Interestingly, although use of pain medications and length of hospitalization were less in the SLAH patients, hospital and surgery costs were significantly greater in the MRIgLITT procedure compared to the open procedure.

More recently, Kang et al. reported the outcome of 20 patients: similar to our series, 8 of 15 (53%, 95% CI 30.1–75.2%) with follow-up at ≥6 months were seizure free after 6 months; one of the non-seizure-free patients had a low-grade glioma in the temporal lobe and arguably should be excluded from this analysis (122). With low numbers, their reported outcome at 1 year was not as good (4 of 11, 36.4%, 95% CI 14.9–64.8%), but 3 of 5 patients at 2 years (60%, 95% CI 22.9–88.4%) were seizure free. An analysis of outcome as a function of ablation volume was non-significant – not surprising, given the small numbers. Patients were discharged home after 1 day (*n* = 17) or 2 days (*n* = 3) with only one patient staying in the intensive care unit (1 day).

The above seizure freedom rates approach but do not match those of the gold-standard open ATL. It is therefore important to note that SLAH does not preclude further surgery. In situations of recurrent seizures in the setting of technical failure to achieve a complete ablation of mesial temporal structures, a repeat SLAH can be performed. This was done in two cases in Willie et al. (99): one did not become seizure free whereas the last patient in that report had been seizure free at 4 months following the repeat ablation, and has remained seizure free subsequently. We have re-ablated nine patients of whom four have become seizure free. In the setting of recurrent seizures after a technically successful SLAH, or repeat SLAH, open resection can be performed. Two patients reported on by Willie et al. (99) underwent ATL, one of whom became seizure free, whereas Kang et al. (122) reported four patients, three of whom became seizure free after ATL. While the mesial structures have been ablated, leaving only some or all of the parahippocampal structures (both ATL and SAH) and other lateral temporal structures (ATL) to be resected, it is important to remove any remnant mesial temporal tissue that may persist. This portion of the operation is challenging due to the disturbed anatomy and requires an in-depth knowledge of mesial temporal anatomy.

##### Complications after SLAH for MTLE

Of the 13 patients reported by Willie et al., complications included one patient with persistent homonymous hemianopia, likely secondary to catheter misplacement, and one patient with a small acute subdural hemorrhage that resolved with evacuation (99). Two of the seven SLAH patients reported by Waseem et al. experienced a partial visual field deficit, and one patient had a postoperative seizure requiring hospital admission (121). One patient had an extended postoperative headache. Of the 20 patients reported by Kang et al., one (the 20th) had a hematoma and edema at the left mesial temporal ablation site and an incongruous superior quadrantanopsia at 2.5 months follow-up (122). One other had a transient 4th nerve palsy with vertical diplopia. Headache was observed in four patients. Notably, one patient with a previous history of chronic depression and suicidal ideation, who was not seizure-free following the ablation, committed suicide 4.4 months following the procedure.

##### Neuropsychological Outcomes after SLAH for MTLE

Drane et al. evaluated the temporal lobe functions of naming and recognition of common nouns and famous persons, functions increasingly being recognized as affected by temporal lobe surgery, in patients following SLAH (*n* = 19) as compared to open resections, including both selective AH and temporal lobectomy (100). Naming and recognition declines were significantly greater in those patients undergoing open resections (32 of 39; dominant: 21 of 22; non-dominant: 11 of 17) as compared to those undergoing SLAH, where none of the 17 patients declined. Kang et al. examined six patients after a median of 10.2 months following SLAH, five of whom had dominant ablations (122). By contrast, there was a statistically significant decline in total learning on the California Verbal Learning Test (CVLT), with three patients – all dominant – considered to have significant decline. Strikingly, there was no change in CVLT delayed recall and no change on the Wechsler Memory Scale (WMS) logical memory (LM) I and II tests, in which up to 37% of patients undergoing ATL would be expected to decline (123). The authors hypothesize that contextual memory tested in the WMS LM task is less affected by SLAH due to the preservation of lateral temporal lobe language processes, as observed by Drane et al. (100), which support semantic aids to memory acquisition in this task. In contrast, the non-contextual processes tested in the CVLT, not aided by semantic context, reveal more the effects of SLAH directly on the hippocampus. Finally, Waseem et al. found clinically meaningful improvement in the delayed verbal memory performance in two of four patients tested, and decline in one of four (121), using the Rey Auditory and Verbal Learning Test. Thus, overall SLAH seems to preserve lateral temporal lobe processes as compared to open surgery – i.e., it minimizes collateral damage – while possibly having less impact on memory as well. Further research to address effects on memory performance is required, as well as the clinical impact of preserved lateral temporal lobe functions.

#### Malformations of Cortical Development and Glial-neuronal Tumors

##### Periventricular Nodular Heterotopia

Laser ablation of periventricular nodular heterotopias (PVH) has been reported in four patients (124–126). Among the three patients with reported follow-up data, one achieved seizure freedom with medication adjustment at 12 months (125), one was seizure free after 8 months (126), and one achieved seizure freedom with subsequent temporal lobectomy and amygdalohippocampectomy (125). Nevertheless, PVHs are surgically difficult to access, and laser ablation provides a useful tool for their treatment in the instances where the PVH, rather than the overlying cortex, is determined to be involved in the seizure onset zone. Laser ablation is particularly useful when functional tissue precludes safe open access to the lesion or its network. In one case, the location was in the posterior frontal region on the dominant side, and the PVH was involved at onset; the patient became seizure free with ablation of the PVH alone, without complication (125). In another case, there were bilateral onsets from PVHs that extended from the periventricular region into the occipital lobes, surrounded by cortical tissue activated by visual fMRI and by optic radiation fibers, as determined by diffusion tensor imaging (126). Laser ablation allowed focal destruction of the PVH bilaterally, with temperature monitoring of surrounding tissue at risk, and the patient became seizure free without visual deficits on field testing. This simply would not have been possible with an open approach.

##### Focal Cortical Dysplasias and Glial–Neuronal Tumors

Focal cortical dysplasias (FCDs) are common surgical targets that are amenable, in many cases, to open surgical resection. There was a single FCD case in Curry et al.’s initial series, with outcome only at 3 months (98). Bandt and Leuthardt noted a patient with a temporal lobe FCD who was seizure free for 18 months following laser ablation with NeuroBlate (127), and Buckley et al. noted a patient with occipital DNET, proven pathologically at the time of the laser ablation, who was seizure free for 6 months (128). The largest series is that of Lewis et al. (129), who reported laser ablation in 17 pediatric patients, including 9 patients with FCD: they included, among these, 2 patients with biopsy-proven gangliogliomas. One difficulty in determining pathologic-related outcomes is that biopsies are either not performed routinely during the LITT procedure, or if they are, they suffer from sampling bias. Five of the nine with FCD did have pathologically proven Palmini type Ia, IIa, or IIb FCD. Of the nine, only three became seizure free at follow-up periods ranging from 12.6 to 35.9 months, and two of these three had pathologically proven gangliogliomas. Thus, the outcome of MRIgLITT for FCDs, as performed by Lewis et al. (129), is substantially worse than from open surgical resections (130), likely exemplifying the empirical observations that complete resection of the seizure onset zone, defined electrographically, is the most important predictor of seizure outcome (131). Perhaps, a more aggressive approach in the future will yield superior outcomes.

Lewis et al. reported complications in four patients, including catheter misplacement in two, edema in one, and cooling failure causing catheter damage in one patient (129). In one of the patients with catheter misplacement, hemorrhage and hydrocephalus required the placement of a temporary drain, after which the hydrocephalus subsequently resolved. Following catheter damage in one patient, ablation was prematurely discontinued, and the catheter tip was left in the parenchyma. Despite these complications, length of stay was just 1 day in 11/17 patients.

#### Tuberous Sclerosis Complex

Curry et al. reported one patient with tuberous sclerosis who was seizure free at 13 months following MRIgLITT (98). Included in the series of Lewis et al. (129) were four patients with definitive tuberous sclerosis, of whom two became seizure-free at 3.5 and 9.6 months, likely too early to draw conclusions. While this pathology seems well suited to focal ablative therapy, as in the case of FCD, it is possible that undertreatment of the lesions in these cases has contributed to the limited results thus far.

#### Cavernous Malformations

In a recent series of five patients with epilepsy caused by cavernous malformations, McCracken et al. found that 4/5 (80%) achieved Engel class I seizure freedom at mean 17.4 months follow-up (132). Importantly, no complications occurred, and length of stay was less than 36 h in all patients.

#### Other Focal Epileptic Lesions

Using the NeuroBlate system, Hawasli and colleagues were able to achieve seizure freedom for over 4 years after ablation of a 5.8 cm^3^ insular focus at an area of a previously silent infarction; the ictal onset zone was identified with depth electrodes (113, 127, 133). The patient had postoperative mild speech and memory deficits requiring cognitive rehabilitation. Similarly, Bandt and Leuthardt ablated an MRI-negative, depth electrode-identified insular focus, achieving seizure freedom for 6 months, with a mild postoperative hemiparesis (127). They also performed laser ablation of the left basal temporal cortex adjacent to an encephalocele concordant with scalp EEG monitoring. In this case, no invasive monitoring was performed, and the patient has been seizure free for 2 years with a single minimally invasive procedure.

## Conclusion

In the treatment of medically refractory mesial temporal lobe epilepsy, minimally invasive techniques have seen both revolutionary and evolutionary changes over the past half century. The development of more precise stereotactic ablation techniques and advances in imaging modalities have provided improved guidance and targeting, while MRI thermometry revolutionized intraoperative monitoring of ablation. The more recent introduction of laser interstitial thermal therapy offers tighter thermal control, and the combination with thermal MRI imaging allows the surgeon to protect critical structures from damage. The use of laser ablation for MTS is only in its infancy, but its superior targeting and intraoperative feedback control makes it an exciting candidate for further investigation. A prospective trial comparing MRI-guided stereotactic laser ablation with open mesial temporal lobectomy would be instrumental in demonstrating the safety and efficacy of this promising new technique for the treatment of epilepsy, but FDA approval of the technology in the US and its widespread availability makes this very difficult, as patient and physician acceptance has been rapid. Other clinical trial approaches will be necessary to demonstrate relative effectiveness and safety with respect to standard open resection techniques. However, it must be considered that the comparison of minimally invasive techniques is not solely to standard open techniques but also to continued medical therapy, as there is a significant number of patients – and referring physicians – who consider the risk, discomfort, or inconvenience of conventional resective surgery preclusive.

## Author Contributions

ML reviewed the literature, extracted data, developed tables, and wrote the manuscript. RG conceptualized the project and reviewed the literature, verified data, and edited and wrote the manuscript.

## Conflict of Interest Statement

RG served as a consultant to Visualase, Inc. and Medtronic, Inc. and received compensation for these services. The terms of this arrangement have been reviewed and approved by Emory University in accordance with its conflict of interest policies. ML declares no conflict of interest.

## Footnotes

^1^The term hippocampotomy will be used to denote ablation, whereas hippocampectomy will be used to denote open resection.
